# Computationally Driven, Quantitative Experiments Discover Genes Required for Mitochondrial Biogenesis

**DOI:** 10.1371/journal.pgen.1000407

**Published:** 2009-03-20

**Authors:** David C. Hess, Chad L. Myers, Curtis Huttenhower, Matthew A. Hibbs, Alicia P. Hayes, Jadine Paw, John J. Clore, Rosa M. Mendoza, Bryan San Luis, Corey Nislow, Guri Giaever, Michael Costanzo, Olga G. Troyanskaya, Amy A. Caudy

**Affiliations:** 1 Lewis-Sigler Institute for Integrative Genomics, Princeton University, Princeton, New Jersey, United States of America; 2 Department of Computer Science, Princeton University, Princeton, New Jersey, United States of America; 3 Department of Computer Science and Engineering, University of Minnesota, Minneapolis, Minnesota, United States of America; 4 Donnelly Centre for Cellular and Biomolecular Research, University of Toronto, Toronto, Ontario, Canada; Stanford University Medical Center, United States of America

## Abstract

Mitochondria are central to many cellular processes including respiration, ion homeostasis, and apoptosis. Using computational predictions combined with traditional quantitative experiments, we have identified 100 proteins whose deficiency alters mitochondrial biogenesis and inheritance in *Saccharomyces cerevisiae*. In addition, we used computational predictions to perform targeted double-mutant analysis detecting another nine genes with synthetic defects in mitochondrial biogenesis. This represents an increase of about 25% over previously known participants. Nearly half of these newly characterized proteins are conserved in mammals, including several orthologs known to be involved in human disease. Mutations in many of these genes demonstrate statistically significant mitochondrial transmission phenotypes more subtle than could be detected by traditional genetic screens or high-throughput techniques, and 47 have not been previously localized to mitochondria. We further characterized a subset of these genes using growth profiling and dual immunofluorescence, which identified genes specifically required for aerobic respiration and an uncharacterized cytoplasmic protein required for normal mitochondrial motility. Our results demonstrate that by leveraging computational analysis to direct quantitative experimental assays, we have characterized mutants with subtle mitochondrial defects whose phenotypes were undetected by high-throughput methods.

## Author Summary

Mitochondria are the proverbial powerhouses of the cell, running the fundamental biochemical processes that produce energy from nutrients using oxygen. These processes are conserved in all eukaryotes, from humans to model organisms such as baker's yeast. In humans, mitochondrial dysfunction plays a role in a variety of diseases, including diabetes, neuromuscular disorders, and aging. In order to better understand fundamental mitochondrial biology, we studied genes involved in mitochondrial biogenesis in the yeast *S. cerevisiae*, discovering over 100 proteins with novel roles in this process. These experiments assigned function to 5% of the genes whose function was not known. In order to achieve this rapid rate of discovery, we developed a system incorporating highly quantitative experimental assays and an integrated, iterative process of computational protein function prediction. Beginning from relatively little prior knowledge, we found that computational predictions achieved about 60% accuracy and rapidly guided our laboratory work towards hundreds of promising candidate genes. Thus, in addition to providing a more thorough understanding of mitochondrial biology, this study establishes a framework for successfully integrating computation and experimentation to drive biological discovery. A companion manuscript, published in *PLoS Computational Biology* (doi:10.1371/journal.pcbi.1000322), discusses observations and conclusions important for the computational community.

## Introduction

In order to understand molecular biology at a systems level, it is first necessary to learn the functions of genes by identifying their participation in specific cellular pathways and processes. While protein sequence and structural analyses can provide valuable insights into the biochemical roles of proteins, it has proven much more difficult to associate proteins with the pathways where they perform these roles. Recently, high-throughput and whole-genome screens have been used to form basic hypotheses of protein participation in biological processes. However, the results of these studies are not individually reliable enough to functionally associate proteins with pathways. Many computational approaches have been developed to integrate data from such high-throughput assays and to generate more reliable predictions [1], but protein function cannot be confidently assigned without rigorous experimental validation targeted specifically to the predicted pathway or process. Surprisingly few follow-up laboratory efforts have been performed on the basis of computational predictions of protein function, and as such, these computational approaches remain largely unproven, and consequently underutilized by the scientific community [2],[3]. Here, we demonstrate that computational predictions can successfully drive the characterization of protein roles using traditional experiments. To test the approach, we systematically measured the mitochondrial transmission rates of a tractable set of *S. cerevisiae* strains carrying deletions of genes predicted to be necessary for this biological process.

The mitochondrion is an organelle central to several key cellular processes including respiration, ion homeostasis, and apoptosis. Proper biogenesis and inheritance of mitochondria is critical for eukaryotes as 1 in 5,000 humans suffers from a mitochondrial disease [4]. *Saccharomyces* has proven to be an invaluable system for studying a variety of human diseases [5],[6], including cancer [7], neurologic disorders [8], and mitochondrial diseases [9]–[11]. Yeast is a particularly attractive model system for studying mitochondrial biology due to its ability to survive without respiration, permitting the characterization of mutants that impair mitochondrial function. The process of mitochondrial biogenesis and inheritance [12] (hereafter, mitochondrial biogenesis) comprises a number of sub-processes that together ensure that new mitochondria are generated and segregated to a daughter cell. Mitochondrial biogenesis begins with the nuclear genes encoding mitochondrial proteins being transcribed, translated, and targeted to the mitochondria for import [13],[14]. The mitochondria must also replicate its own genome [15] and assemble the numerous membrane-bound complexes necessary for proper function [16]. During mitochondrial transmission, the mitochondria are actively transported along actin cables to the bud neck, where they are then segregated between the mother and daughter cells [17]. In addition to the experimental utility of yeast, it is well suited for the application of computational prediction approaches due to the availability of manually-curated annotations of yeast biology and the available wealth of genome-scale data.

Previous efforts have focused on identifying mitochondria-localized proteins through laboratory techniques such as mass spectrometry and 2D-PAGE [18],[19] and through computational predictions of cellular localization [20],[21]. These approaches have resulted in the identification of over 1,000 mitochondria-localized proteins in *S. cerevisiae* [22]. However, despite yeast's convenience as a model system, mitochondrial phenotypes of ∼370 of these 1,000 localized proteins have not been characterized, so the mitochondrial role of these predictions is unknown (over half of these 370 have no known function in any cellular process). Previous computational efforts have attempted to address this problem by predicting putative mitochondrial protein modules [20] and examining expression neighborhoods around mitochondrial proteins [23]. While valuable, these predictions of protein function have not been confirmed through laboratory efforts. Rather, these studies have performed assays for protein localization to the mitochondria, which is not sufficient to convert these predictions to concrete knowledge of protein roles [24].

Here, we describe a strategy that combines computational prediction methods with quantitative experimental validation in an iterative framework. Using this approach, we identify new genes with roles in the specific process of mitochondrial biogenesis by directly measuring the ability of cells carrying deletions of candidate genes to propagate functioning mitochondria to daughter cells. We assayed our 193 strongest predictions with no previous experimental literature evidence of phenotypes and interactions establishing a function in mitochondrial biogenesis. By these assays we experimentally discovered an additional 109 proteins required for proper mitochondrial biogenesis at a level of rigor acceptable for function annotation. Further, we identified more specific roles in mitochondrial biogenesis for several predicted genes through mitochondrial motility assays and measurements of respiratory growth rates. We also discovered genes with redundant mitochondrial biogenesis roles through targeted examination of double knockout phenotypes. This demonstrates that using an ensemble of computational function prediction methods to target definitive, time-consuming experiments to a tractably sized set of candidate proteins can result in the rapid discovery of new functional roles for proteins. Our results also show that most mutants resulting in severe respiratory defects have already been discovered. This is likely to be the case for mutant screens in many fundamental biological processes, because saturating screens have discovered mutations with strong phenotypes. However, even in a well-studied eukaryote like *S. cerevisiae*, there are many processes that have not yet been fully characterized by identifying all proteins required for its normal function [25]. As such, most of the remaining undiscovered protein functions are only identifiable by rigorous, quantitative assays that can detect subtle phenotypes, such as those used by our study.

## Results

**Figure 1:**
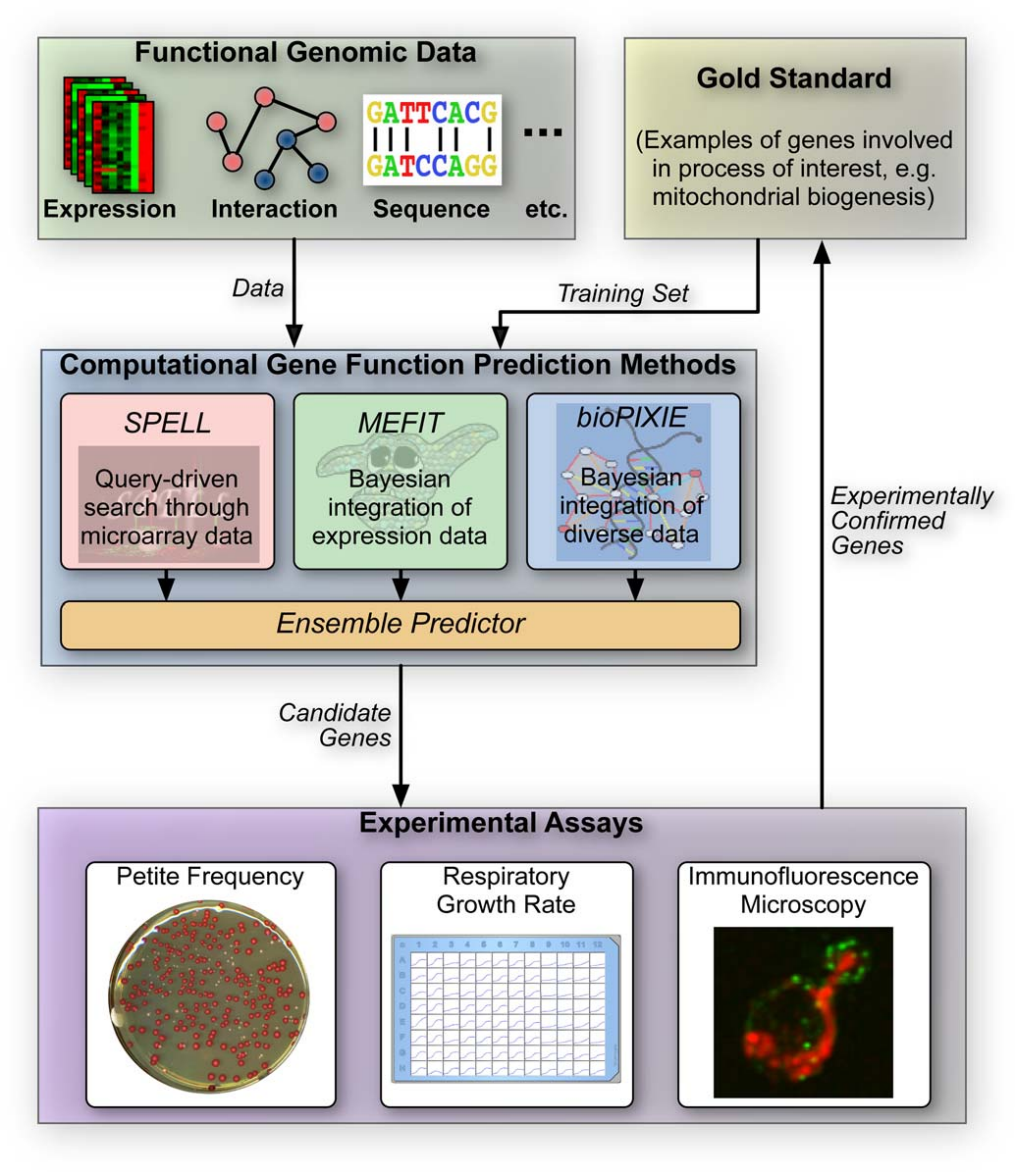
An overview of our iterative framework integrating computational and experimental methodologies for discovery of gene function. Our study uses an ensemble of computational gene function prediction methods (bioPIXIE [26], MEFIT [28], and SPELL [29]), each of which predicts new genes involved in mitochondrial function based on high-throughput data and examples of known mitochondrial proteins (the gold standard, Table S3). Complete lists of predictions are provided (Tables S4 and S5). We selected test candidates by integrating these approaches based on estimated precision of each method and tested these predictions experimentally using three biological assays (see Methods for details). Upon evaluating these experimental results, the proteins newly discovered to be involved in mitochondrial function were added to the known examples, and the process was iterated to comprehensively characterize additional mitochondrial proteins. See Table 1 for an overview of our results, and Table S2 for a full listing of results.

We utilized an ensemble of computational gene function prediction approaches to systematically identify candidates for involvement in mitochondrial biogenesis. These candidates were experimentally assayed, and the confirmed predictions were then utilized as inputs for a second round of prediction and experimentation. A schematic overview of this approach is shown in Figure 1.

### An Ensemble of Computational Function Prediction Methods Was Used to Iteratively Target Experiments

We trained an ensemble of three computational prediction methods (bioPIXIE [26],[27], MEFIT [28], and SPELL [29]) using genomic data that we collected from many sources (full list in supplementary materials) and a set of 106 genes known to be involved in mitochondrial organization and biogenesis based on published experiments as curated by the *Saccharomyces* Genome Database (SGD) [30]. Genes are assigned by SGD to this biological process if published experiments have definitively demonstrated functions involved in the formation, assembly, or disassembly of a mitochondrion. The classification of mitochondrial organization and biogenesis includes genes that affect mitochondrial morphology and distribution, replication of the mitochondrial genome, and synthesis of new mitochondrial components.

An intuitive description of our computational methods is that each employs “guilt by association” to identify genes exhibiting similar data patterns to the genes used for training (further details in Methods). The ensemble was used to rank all genes in the genome from most likely to be involved in mitochondrial biogenesis to least likely. We selected the top 183 most confident genes that were not included in the training set for experimental validation. Of these, we found existing experimental literature evidence of involvement in mitochondrial biogenesis for 42 proteins, and as such we included these in our set of positive controls (along with 6 genes from the training set). The remaining 141 proteins comprised our set of first iteration predictions, as none of these proteins appeared in published experiments that demonstrated their requirement for proper mitochondrial biogenesis. We assayed these predicted genes experimentally as described below. We then augmented our training set of genes known to be involved in mitochondrial biogenesis with the experimentally verified predictions (using both our experiments and the uncurated published literature, see methods) and repeated this process to generate a second iteration of predictions. From this second iteration, we selected the 52 most confident predictions that were not previously tested and performed the same experimental assays.

### Petite Frequency Assay Quantitatively Detected Defects in Mitochondrial Biogenesis

In order to confirm the potential roles of our candidate genes in mitochondrial biogenesis, we employed an experimental assay that measures the rate of generation of cells lacking respiratory competent mitochondria (called “petite” cells [31]). This assay reliably detects defects in mitochondrial biogenesis, but it is too time consuming to perform on a whole genome scale. Wild-type yeast from the S288C genetic background produce petite daughter cells at a baseline frequency of ∼20% [32], but mutation of genes involved in mitochondrial biogenesis can significantly alter this rate.

**Figure 2:**
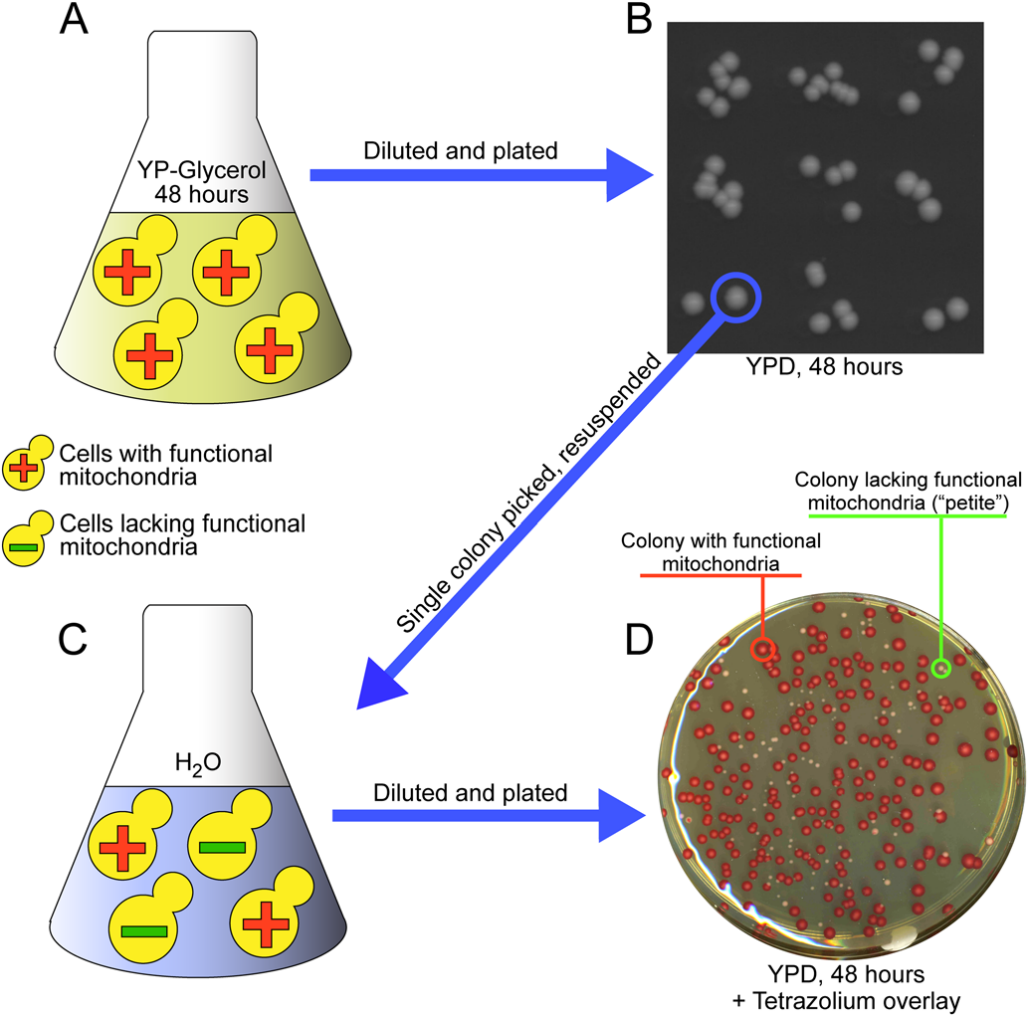
Schematic overview of the petite frequency assay. (A) Initially, strains were grown in a non-fermentable carbon source (liquid YP-Glycerol) for 48 hours. All cells growing under this condition must be respiratory competent and contain functional mitochondria. Any strain with no viable cells after this step was deemed respiratory deficient and did not continue in the assay. (B) Cell cultures were serially diluted and plated on a fermentable medium (YPD) and grown for 48 hours to form colonies founded from a single cell. At this point, the requirement for respiratory competency is lifted, so that daughter cells can survive while losing respiratory function. (C) A single colony is picked and briefly re-suspended in water. (D) The suspension is diluted and plated on YPD and grown to form colonies founded from single cells. After 48 hours, agar containing tetrazolium is overlaid on the plates. Colonies founded by respiratory competent cells will take up the tetrazolium and appear large and red. Colonies founded from respiratory deficient cells (“petite” colonies) appear smaller and white.

We measured the frequency of petite formation for single gene deletion strains of all 193 candidate genes (141 from the first iteration, 52 from the second) and for 48 positive control genes. To reduce the effects of suppressor mutations and aneuploidy associated with the yeast deletion collection [33], we sporulated the heterozygous Magic Marker deletion collection [34] and isolated six independent haploid deletion mutants for every gene tested. Individual deletion strains were grown in media requiring aerobic respiration for growth (glycerol), and strains completely unable to grow were deemed respiratory deficient and did not continue in the assay. The remaining mutants were then assayed by measuring the ratio of petite cells to total cells in a colony founded from a single cell. At least twelve matched wild-type sporulation isolates were assayed on each day of experiments in order to establish baseline frequencies. For each gene tested, petite frequencies were measured for at least eight colonies and compared to the distribution of wild-type frequencies measured in parallel on each day of experiments, which allowed us to quantitatively detect subtle phenotypes with statistical rigor. A schematic of this assay is shown in Figure 2 and further details are available in Methods.

### Computationally-Driven Experimentation Discovered a New Role in Mitochondrial Biogenesis for 109 Proteins

**Figure 3:**
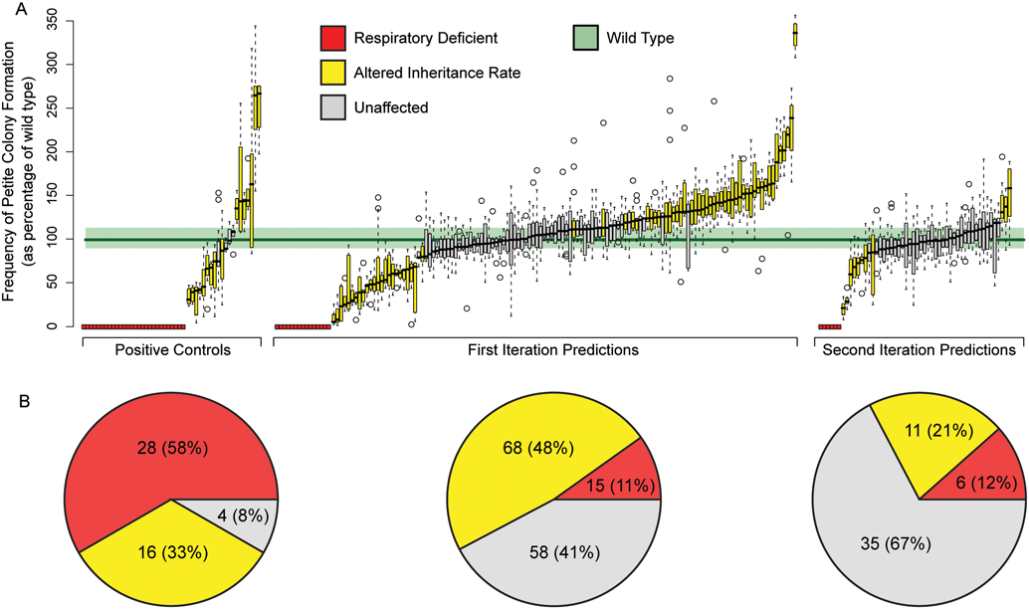
The combination of computational predictions and quantitative assays discovers novel genes involved in mitochondrial function. (A) Mitochondrial transmission rates of single gene knockouts were determined for 193 genes predicted to be involved in mitochondrial function and for 48 control genes known to be involved (raw data in Table S6). A box plot is shown for each deletion strain tested; red indicates the inability to grow on a non-fermentable carbon source (glycerol), yellow indicates a mitochondrial transmission rate significantly altered from wild type, and gray indicates no significant difference from wild type. Significance was determined using a Mann-Whitney U-test comparing at least 12 independent measurements of wild type to at least 8 independent measurements of each mutant strain. The green shaded region indicates one quartile above and below the median rate for all 358 wild type replicates. A total of 100 of the 193 prediction candidates were confirmed (an additional 9 genes were confirmed through double knockout analysis, see Figure 4). (B) Distribution of petite frequency phenotypes among positive controls (left), first iteration predictions (center), and second iteration predictions (right) with colors as in (A). Severe phenotypes (red) were more prevalent among positive controls, while the majority of confirmed predictions exhibited an intermediate phenotype (yellow). We hypothesize that this difference is due to a bias towards detection of extreme phenotypes in classical genetic screens and high throughput methodologies.

In our first iteration of prediction and experimental testing, 83 of our initial 141 predictions (59%) were confirmed to play a role in mitochondrial biogenesis as they exhibited a significantly altered petite frequency rate compared to the wild-type distribution (FDR corrected Mann Whitney U-test p-value <0.05; see Figure 3A). These 83 newly confirmed predictions were added to the training set, and we then performed another iteration of prediction and experimentation. In this second iteration, 17 of the 52 predictions (33%) were experimentally confirmed (Figure 3A). Based on the second iteration predictions, we also examined a targeted set of double knockout mutants and experimentally confirmed 9 more proteins that exhibit synthetic petite frequency defects (full details below). Further, the petite frequency assay demonstrated a high level of sensitivity as 44 of our 48 positive controls (92%) exhibited a significant phenotype (the remaining 4 are discussed further below). All together, after both iterations of our approach we discovered a role in mitochondrial biogenesis by demonstrating significant phenotypic alterations for 109 of our 193 (56%) total predictions (see Table 1 for breakdown).

**Table 1:** Summary of results.

	Number Selected for Testing	Number with Significant Mitochondrial Transmission Phenotype
Positive Controls	48	44
First Iteration Predictions	141	83
Second Iteration Predictions	52	17
Synthetic Interaction Predictions	26	9

We iteratively employed an ensemble of computational function prediction methods to select candidate genes for experimental testing. Confirmed predictions from the first iteration were added to the training set for the second iteration. Promising candidates for synthetic interactions were also selected after our second iteration for testing with double mutant assays.

These newly characterized functions include 42 genes with other previously known functions (not in mitochondrial biogenesis) and 68 genes with no previously characterized cellular role. For example, we observe that mutation in the functionally uncharacterized *TOM71* causes a 44% increase in petite frequency. While Tom71 has been co-localized with the translocase complex responsible for protein import through the mitochondrial outer membrane, previous work (largely *in vitro*) has not identified a strong functional defect associated with Tom71 in translocase activity [35]. Our confirmation that *TOM71* significantly affects mitochondrial transmission rates strongly suggests that it does indeed play a role in mitochondrial import, at least for some subset of proteins required for mitochondrial inheritance or biogenesis. The identification of a functional role for 68 previously uncharacterized proteins is particularly striking as this covers roughly 1 in 18 of the remaining ∼1200 proteins in yeast that still have no known functional role [25].

### Subtle Phenotypes Are Predominant Among our New Discoveries

We observed a striking difference in the severity of petite frequency phenotypes in single gene knockouts between the confirmed gene predictions and the positive controls (Figure 3B). Of the 44 positive controls demonstrating a significant phenotype, the majority exhibited a complete loss of respiratory function (28 of 44, 64%) as opposed to the more subtle phenotype of altered mitochondrial transmission (16 of 44, 36%). The proportions of subtle and severe phenotypes were reversed in our predictions experimentally confirmed by single gene knockouts, in which 79 of 100 mutants (79%) showed altered mitochondrial transmission while only 21 of 100 mutants (21%) were respiratory deficient. The quantitative nature of these phenotypes among our novel discoveries may indicate why they have not been previously associated with mitochondrial biogenesis by either classical genetic screens or high-throughput techniques [24],[36], which generally assay extreme rather than subtle phenotypes. In further support of this observation, since undertaking this study, 8 of our 100 confirmed candidates have been associated by other groups to mitochondrial biogenesis (*COA1* [37], *IBA57* [38], *GUF1* [39], *ATP25* [40], *QRI5* [41], *GRX5* [42], *REX2* [43], *RMD9* [44]), and 4 of these 8 exhibited the most extreme phenotype of respiratory deficiency in our study (Table S2).

### Computational Iteration Identifies Candidates with Redundant Mitochondrial Function Verified through Double-Mutant Analysis

The confirmation rate from our second iteration decreased from our first iteration (59% to 33%), which suggests we may be nearing the limit of predicted genes that can be verified using the single knockout petite frequency assay. In particular, examining single gene deletion strains prohibits characterization of the roles of redundant proteins or genes that only exhibit synthetic phenotypes. In fact, all four of our 48 positive controls that did not exhibit a significant petite frequency phenotype are known to synthetically interact with at least one other gene involved in mitochondrial biogenesis and inheritance [45]–[48]. Our second iteration prediction results indicate which of our unconfirmed predictions are worthy of further investigation with double mutant analysis or additional assays, particularly in light of additional localization evidence. Following the second round of computational prediction, 26 of the 58 initially unconfirmed predictions persisted as highly ranked candidate genes while the remainder decreased in confidence. Of these, 22 (85%, hypergeometric p-value <10^−9^) candidates are known to localize to the mitochondria, while only 1 of the remaining 32 unconfirmed candidates (3%) is similarly localized.

**Figure 4:**
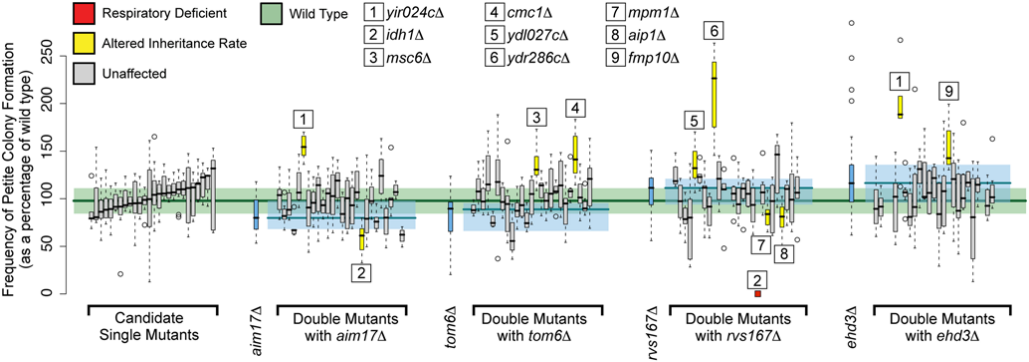
Double mutant petite frequency phenotypes. Based on their persistence as strongly predicted candidates during our second iteration, we selected 26 genes unconfirmed by single mutant analysis for investigation of synthetic phenotypes. The single mutant petite frequency is shown for each of these strains on the left. Each of the 26 strains was crossed with 4 genes known to be involved in mitochondrial transmission (*aim17*Δ, *tom6*Δ, *rvs167*Δ, and *ehd3*Δ) to create ∼100 double mutant strains. Results are shown in blue for each of the 4 strains crossed into, followed by all 26 double mutants constructed against that strain (raw data Table S7). The order of the double mutants is the same as in the 26 single mutants shown on the left. Colors are as in Figure 3. Significantly altered double mutant strains are marked with numbers, corresponding to the key above the box plots.

To test the hypothesis that these 26 high-confidence unconfirmed predictions represented genes that had redundant mitochondrial function, we performed targeted double mutant analysis looking for synthetic interactions. We chose 4 deletion mutants (*aim17*Δ, *rvs167*Δ, *tom6*Δ and *ehd3*Δ) confirmed to be involved in mitochondrial biogenesis with modest petite frequency phenotypes to cross with these 26 candidates. Choosing mutants with modest phenotypes was necessary to allow for a strong synthetic interaction to be observed. We tested 99 double mutant strains and observed 11 significant synthetic phenotypes (FDR corrected Wilcoxon rank-sum p-value <0.05) spanning 9 of 26 mutants that did not display a single mutant phenotype (Figure 4). While some of our double mutants exhibit suppression, we did not focus on these interactions because of the modest nature of the single mutant phenotypes. Instead we focused on synthetic defects which we could rigorously define as the double mutant petite frequency being significantly different from both single mutants and the wild-type petite frequency. Of the genes exhibiting significant double mutant phenotypes, 1 was synthetic respiratory deficient and 8 demonstrated altered petite frequency. The 9 genes showed a specific pattern of synthetic phenotypes, as 7 interacted with only 1 of the 4 known mitochondrial biogenesis genes used to generate double mutants. These specific synthetic interactions suggest the functions these genes may perform in mitochondrial biogenesis. For example, the four genes (*AIP1*, *MPM1*, *YDL027C*, and *YDR286C*) that specifically interact with *rvs167*Δ are potentially involved in the actin-based transmission of mitochondria to the daughter cell as Rvs167 is a regulator of actin polymerization [49]. In fact, the only known actin-localized protein among our 26 candidates, Aip1, had a genetic interaction only with the *rvs167*Δ(Figure 4).

The high rate of synthetic phenotype recovery (9 out of 26 candidates tested) was made possible by the use of computation to limit the number of double mutants queried. There were 58 unconfirmed predictions from the first round of our analysis, and 95 genes tested in this study have the quantitative petite frequency phenotypes necessary for double mutant analysis. Combining these 95 confirmed genes with the 58 unconfirmed genes yields 5,510 possible double mutants to assay, which is far too large to reasonably test with the quantitative petite frequency assay. However, we used computation in two ways to reduce the number of double mutants screened to ∼100. First, we used computational iteration to identify the subset of unconfirmed predictions most likely to be involved in mitochondrial biogenesis. Second, we used the functional networks generated by the bioPIXIE algorithm [26] to select four genes from different sub-functions in mitochondrial biogenesis. This allowed us to test less than 2% of the possible double mutants, but still identify phenotypes for 9 of 26 candidates (35%) due to the efficiency of our computational approach.

### Computationally Targeted Experiments Characterize New Protein Functions Regardless of Known Localization

While we expect high correlation between localization to the mitochondria and involvement in mitochondrial biogenesis, many proteins not localized to mitochondria are vital for regulating mitochondrial function and biogenesis [17]. Thus, a candidate gene approach based solely on protein localization would neglect many important participants in normal mitochondrial biogenesis. Our use of computational predictions to drive experimental discovery is unbiased with respect to any one genomic feature or assay. In this study, 47 (43%) of our 109 newly confirmed discoveries are not known to localize to the mitochondria [30],[50] and would have been overlooked in a screen of mitochondria-localized proteins lacking known functions. Further, the accuracy of our predictions for non-mitochondria-localized proteins is comparable to that for mitochondria-localized proteins (44% vs. 59%, respectively). Thus, computational predictions can broaden the scope of potential discoveries beyond a more restricted candidate gene approach based on a single experimental technique or data source.

### *AIM21* Is Required for Proper Mitochondrial Motility

Specific examples of non-mitochondria-localized proteins critical for mitochondrial biogenesis include proteins linking mitochondria to the actin cytoskeleton. Several of our novel discoveries have literature evidence associating them to the actin cytoskeleton but no evidence suggesting a role in mitochondrial transmission [51]–[53]. One of these genes, the uncharacterized ORF *YIR003W* (*AIM21*), has been shown to co-localize with actin in high-throughput studies [51] and was predicted as an interactor with the actin cytoskeleton with high confidence by our system bioPIXIE [27]. We found that strains carrying a deletion of *YIR003W* grow normally on glycerol but form petites at a frequency of 166% of wild type cells, one of the highest petite frequencies observed in our experiments.

**Figure 5:**
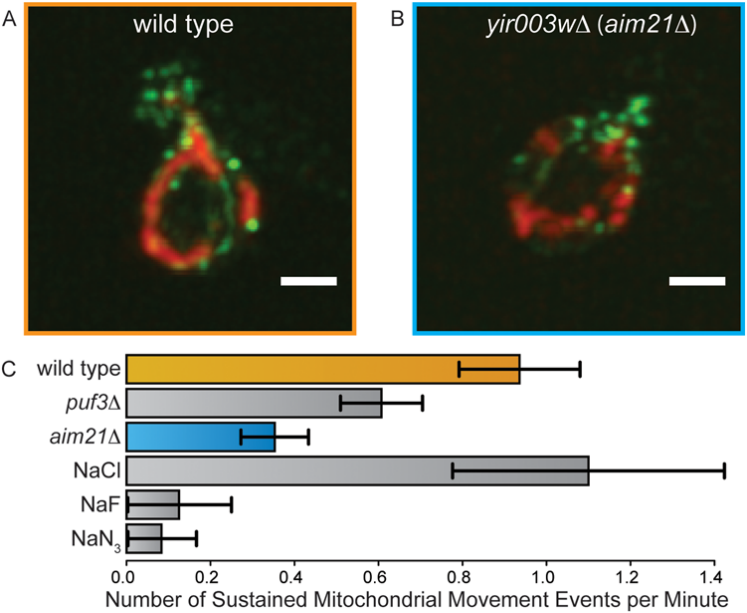
*YIR003W* (*AIM21*) is required for mitochondrial motility. (A)–(B). Dual immunofluorescence of mitochondria (outer membrane protein porin stained in red) and actin (total actin, stained in green) in the indicated yeast strains (scale bar 2 µm). (C) Mitochondrial motility was measured in strains carrying an integrated mitochondrially-targeted GFP (methods) by tracking the movement of the tip of a mitochondrion within a budding cell every second for two minutes. A sustained mitochondrial movement is defined as movement in the same direction for at least three consecutive seconds. *PUF3* is a gene with known involvement in mitochondrial motility [54]. To determine the frequency of sustained mitochondrial movement resulting from Brownian motion or other passive processes (methods), sustained mitochondrial movement was measured in the presence of the metabolic inhibitors sodium azide (NaN3) and sodium fluoride (NaF). 10 mM concentrations of these inhibitors were compared to a control of 10 mM sodium chloride (NaCl). Raw data are available in Table S8. Due to its lack of static actin or mitochondrial phenotypes, the motility defect in *AIM21* mutants would be difficult to find without integrative computational predictions driving specific experimental assays.

To better understand the mitochondrial transmission defect in this mutant, we used our computational predictions to direct experiments targeting the role of the actin cytoskeleton in mitochondrial transmission. The morphology of the actin cytoskeleton and of the mitochondria in this mutant was visualized by dual immunofluorescence (Figure 5A,B). In the *yir003w*Δ mutants, the actin skeleton appears relatively normal, with typical polarization of actin patches toward the daughter (Figure S1), and the mitochondria show no gross structural perturbation in these mutants. However, by observing sustained mitochondrial movement events, we assessed mitochondrial motility for this mutant and found severe defects comparable to a *puf3*Δ strain (Figure 5C), a gene known to be involved in mitochondrial motility [54]. Even though this mutant displayed no overt morphological phenotypes, detailed analysis of *YIR003W* uncovered a more subtle, specific defect in mitochondrial motility.

### Characterization of Predicted Genes with Respiratory Growth Assays

**Figure 6:**
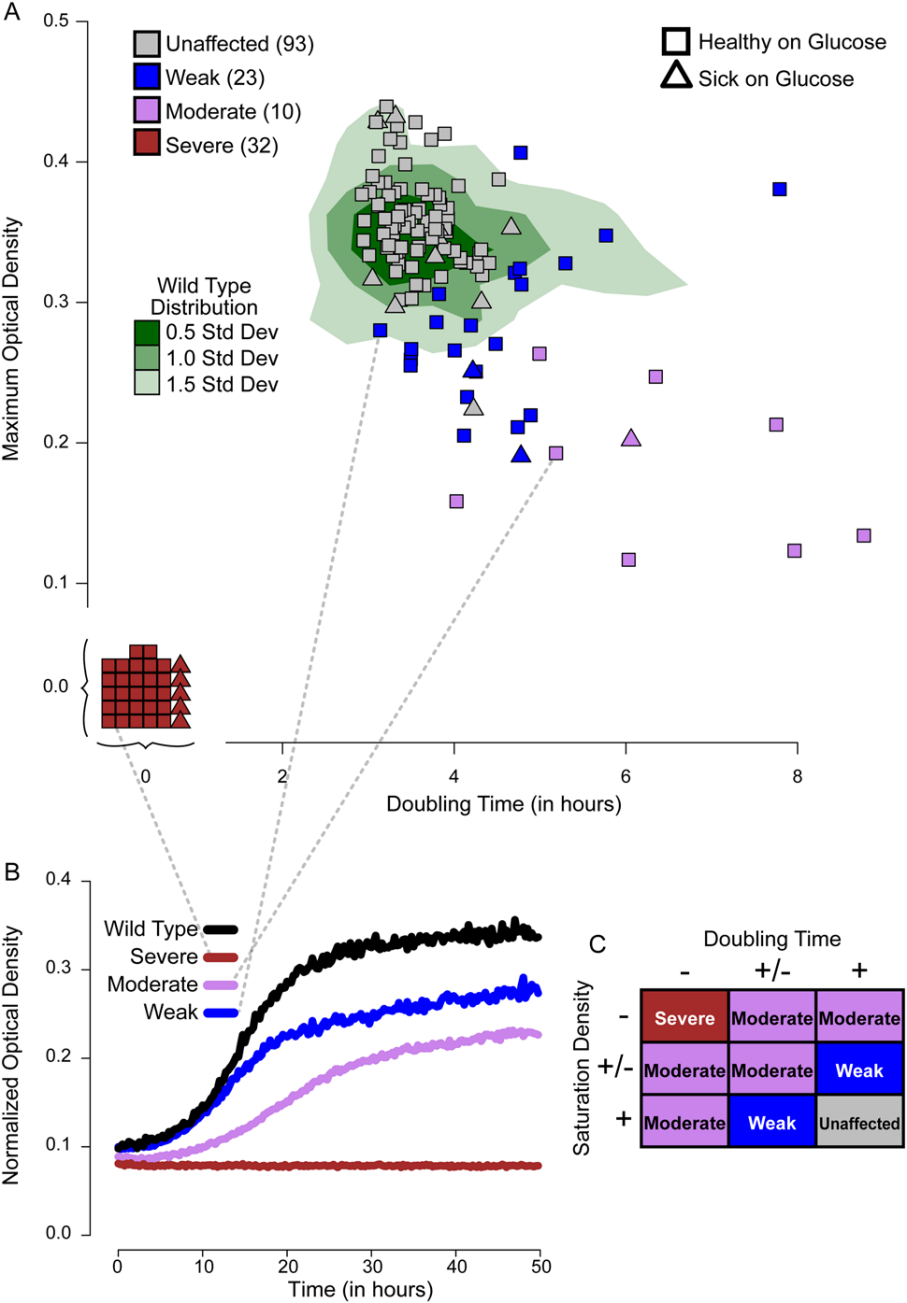
Respiratory growth phenotypes. (A) Scatter plot of growth rate and saturating density measured from growth curves in minimal non-fermentable media (raw data in Dataset S1). The vertical axis indicates the maximum (saturating) optical density achieved by the strain, and the horizontal axis represents the estimated doubling time based on an exponential fit to the growth curve (methods). Green shading indicates the distribution of all 536 wild type measurements. Triangles represent strains with saturation density and/or doubling time significantly altered on glucose, while squares represent strains that showed normal growth on glucose. Each point is colored by the strength of its respiratory growth phenotype (see part C). (B) Example growth curves for wild type and strains representing each of the three phenotypic classes: weak, moderate, and severe respiratory growth defects. (C) Determination of respiratory growth phenotype. Each growth parameter (saturation density and doubling time) was statistically scored as no effect (+), intermediate effect (+/−), or extreme effect (−) (methods). The combination of saturation density and doubling time results produces a final respiratory growth phenotype, with maroon representing a severe defect, purple a moderate defect, blue a weak defect, and gray no defect. Respiratory growth is not strongly correlated with petite frequency (Figure S3).

To further characterize our predictions, we assayed single gene knockout mutants for respiratory growth defects, as assembly of the complexes required for respiration is a critical step in mitochondrial biogenesis. We quantitatively measured growth profiles of most of our single gene deletion mutants under respiratory growth conditions (glycerol) comparing them to growth in fermentative conditions (glucose) as a control. A 96-well plate incubator and optical density reader was used to determine growth profiles for six independent replicates of each deletion strain tested and for two matched wild-type isolates of each strain (24 control wells per plate, see Methods for details). Exponential growth rates and saturation densities were calculated for each strain (Figure 6A), and both of these parameters were assessed for statistical significance relative to the distribution of all wild-type controls. Significant phenotypes were only reported if the defect was unique to the glycerol growth condition (i.e. was not present in the glucose growth curve) in order to ensure that the growth defect is respiration specific. By combining the growth rates and saturation densities (Figure 6C), we arrived at a respiratory growth phenotype that classifies each mutant as severe, moderate, weak, or unaffected. An example growth curve of each class is shown in Figure 6B.

As expected, nearly all mutants classified as respiratory deficient in the petite frequency assay were classified as severely defective in the respiratory growth assay. However, we also observed significant respiratory growth phenotypes for 29 mutants without previously reported respiratory impairments in the literature. Of these, 22 exhibited a weak or moderate defect that may have been difficult to observe in whole-genome screens assaying respiratory growth [55],[56]; the remaining 7 severe phenotypes might have been previously overlooked due to suppressor mutations in the systematic deletion collection. While employing multiple replicates in such assays lowers overall throughput, these results suggest that testing many replicates enables more complete discovery of subtle respiratory growth phenotypes.

### Computationally Directing Experimental Efforts Can Accelerate Discovery Rates

We employed thorough assays performed in replicate in order to detect important but subtle phenotypic variations. As such, it is impractical to scale these assays to the entire genome at the same level of rigor. In fact, given our rate of experimental efforts, it would require nearly 7 years for us to apply the petite frequency assay to all viable single gene deletion strains. However, by using computational predictions of protein function as a form of initial genetic screen, we were able to target our efforts towards the most promising candidates first. This is important for testing single gene deletions, but it is imperative for assaying potential synthetic defects. There are 18 million possible double gene knockouts in *S. cerevisiae*, a number far too large to comprehensively test for a broad range of phenotypes. However, we were able to discover 11 synthetic mitochondrial biogenesis defects by assaying a small, computationally chosen fraction of this available space. In all, by utilizing computational predictions of proteins involved in mitochondrial biogenesis, we have rapidly characterized new functional roles for 109 genes.

## Discussion

We have used computational predictions of gene function to direct focused, non-high-throughput laboratory experiments, confirming 109 proteins required for normal mitochondrial biogenesis in *S. cerevisiae*. These discoveries include 68 genes with no previously known function (5% of the remaining ∼1,200 uncharacterized *S. cerevisiae* genes) and 47 proteins not currently known to localize to the mitochondria. For several genes, our results provide evidence of involvement in specific sub-processes of mitochondrial biogenesis (e.g. *AIM21*/*YIR003W* in mitochondrial motility). No previous study has systematically tested computational predictions of protein functions using non-high-throughput laboratory techniques; the 56% accuracy established by our study demonstrates the potential of such computationally driven genetic investigations for direct future biological discoveries. In addition to the biological discussion presented here, this study resulted in several observations and conclusions important for the computational community, which are discussed in a companion manuscript [57]. Of our newly characterized mitochondrial genes, 53 have strictly defined human orthologs, 5 of which are associated with known diseases (see Methods).

### Computation Identifies Subtle Phenotypes Confirmed by Experimentation

Computational function prediction and non-high-throughput laboratory experiments complement each other in another important way highlighted by these results: the combination of these two techniques can rapidly identify subtle, quantitative phenotypes that are difficult to detect with high-throughput assays. When investigating well-studied processes (such as mitochondrial biology), most genes for which loss of function completely disrupts the process have already been discovered, since such extreme phenotypes are relatively easy to detect. This is evidenced by the strong enrichment for severe phenotypes among our positive control set. Many important biological functions also tend to be redundant, such that disruption of a single gene results in only a mild (but quantifiable) perturbation of the process rather than loss of function. This is likely to be even more prevalent in higher organisms, which employ far more redundancy than does *S. cerevisiae*, and it is also key to understanding the molecular mechanisms of many diseases. Deletion of yeast orthologs of human mitochondrial disease genes is significantly more likely to cause a modest respiratory growth defect than a severe defect [36]; similarly, since aerobic respiration is essential for mammalian viability, many disease-related mutations are unlikely to completely disrupt human mitochondrial function. Rather, these mutations tend to cause diseases by partially compromising the mitochondria [24]. Recently, Fan *et al.* [58] compared several mouse models of mitochondrial disease, and found that subtle mutations caused disease in adult animals, while more severe mutations were suppressed at a high frequency. Subtle mitochondrial defects accrued over time have also been of increasing recent interest as related to aging in human beings [59]. As the field continues to investigate the molecular basis of human disease and aging, the relationship between diseases and mutations incurring subtle functional perturbations is likely to extend far beyond mitochondrial biology.

### Computational Approaches Quickly Provide Accurate, Unbiased Predictions of Protein Function

Using computational techniques to generate candidate gene lists for further investigation has several advantages relative to individual high-throughput experimental screens, with comparable accuracy. First, computational data integration has the capacity to take advantage of large collections of existing publicly available experimental data; this can reveal information on a process of interest (e.g. mitochondrial function) by simultaneously examining many previous results. Additionally, computational predictions can often be generated in days or weeks, in contrast to the months or years required to conduct many traditional experimental assays. Computational integration of multiple data sources can also be less biased to any one biological feature of the candidate genes. For example, high-throughput localization studies have identified hundreds of mitochondrial genes without known functions [22],[50], but this approach would have missed the 51 genes (∼50%) discovered in this study that do not have known mitochondrial localization. This lack of bias assisted us in discovering functions for 68 of the uncharacterized genes in *S. cerevisiae*, all of which represent healthy and viable mutants in the yeast deletion collection with no extreme single mutant phenotype detected by previous screens. Thus, while genetic screens are important and valuable for candidate selection, computational prediction approaches integrating existing data are a viable, accurate alternative, particularly in areas with prior knowledge.

### Mitochondrial Phenotypes Are Unlikely to Represent Pleiotropic Effects

The 51 genes we confirm to be necessary for mitochondrial biogenesis that have no known mitochondrial localization raise the possibility that these mutants are somehow indirectly affecting biogenesis. Several lines of evidence argue against this possibility. First, we expect that many of these 51 proteins will localize to specific cellular structures controlling biogenesis outside of the mitochondria. For example, 13 of the 51 are known to localize to actin cytoskeleton and/or the bud neck, both structures that play intimate roles in mitochondrial transmission. Of the remaining 38 proteins, 3 were computationally predicted to localize to the mitochondria by another study [50], 11 have no known localization, and 7 have only been localized to the cytoplasm by high-throughput microscopy (which does not exclude mitochondrial localization). Further study of these 38 proteins may identify as-yet-undiscovered mitochondrial localization or highlight the importance of other cellular processes necessary for mitochondrial biogenesis (e.g. transcriptional regulation of nuclear-encoded mitochondrial genes).

### Decreased Petite Frequency Identifies Petite Negative Mutants

Among our deletion strains exhibiting the subtle phenotype of altered petite frequency, we observed mutants with both statistically significant increases and decreases in frequency. Increased petite formation clearly indicates a failure in normal mitochondrial biogenesis or transmission. One possible explanation for a decreased petite frequency is a distinct phenotype referred to as “petite negative” [60]. Petite negative mutants display synthetic lethality or sickness with respiratory deficiency, which impairs the survival of petite cells and thus decreases their frequency. Known petite negative mutations occur in mitochondria-localized proteins that normally support the maintenance of the mitochondrial membrane potential in the absence of respiration [60]. Decreased petite frequency was observed in nine (19%) of our positive controls, two of which (*FMC1* and *PHB1*) are known petite negative mutants [61]. Previously, traditional genetics and genome-wide screens have identified 21 petite negative mutations that result in synthetic lethality [61]. Among our 100 discoveries in mitochondrial biogenesis from single gene knockouts, we found 32 additional mutants exhibiting a decreased petite frequency indicative of non-lethal synthetic interactions. Many of the characterized petite negative genes have roles in the assembly and turnover of ATP synthase complexes, and so these genes may be a rich target for further study [61].

### Extensions to Specific Mitochondrial Sub-Processes

While additional work will be necessary to associate all of the proteins discovered in this study with specific sub-processes (such as mitochondrial genome maintenance, mitochondrial protein import and mitochondrial complex assembly), we have already identified two groups with interesting potential responsibilities in mitochondrial biogenesis. The first group is identified by comparing our glycerol growth rate data with our petite frequency results. Mitochondrial biogenesis and respiratory growth are partially overlapping processes that intersect in the translation and assembly of respiration complexes. As such, 55 of the 67 assayed mutants (82%) that exhibited an altered petite formation frequency had only weak or unaffected phenotypes in the respiratory growth assay (Table 2). The remaining 12 mutants exhibiting altered transmission rates were classified as either severe or moderate in the respiratory growth assay, thus, these mutants demonstrate both an transmission defect and a strong defect in respiration. These include four positive controls (*CIT1*, *COX14*, *FMC1*, and *MRP49*) known to be directly involved in the translation and assembly of respiratory complexes [62]–[65]. Additionally, since the beginning of this study, two of the eight additional genes in this class (*MAM33* and *COA1*) have been shown to function in aerobic respiration [37],[66],[67]. This suggests that the remaining six genes newly characterized by this study (*AIM8*, *AIM23*, *AIM24*, *AIM34*, *CTK3*, and *UBX4*) are also functioning in the assembly of respiration complexes. Though the components of the mitochondrial complexes that generate ATP have been identified for some time in yeast, extensive chaperone, assembly, and turnover machinery for these complexes remains to be fully elucidated. The assembly and maintenance of these respiratory complexes is thus a likely role for these 8 proteins.

**Table 2:** Overlap between petite frequency and respiratory growth phenotypes.

		Petite Frequency Phenotype		
		Respiratory Deficient	Altered Transmission Rate	Unaffected
Respiratory Growth Phenotype	Severe	28	4	0
	Moderate	0	8	2
	Weak	2	12	9
	Unaffected	0	43	50

We observe that the majority of single deletion strains deemed respiratory deficient in our petite frequency assay exhibited severe respiratory growth defects as well. Interestingly, 12 mutants with altered mitochondrial transmission rates exhibited either severe or moderate respiratory growth defects, indicating that these genes may be involved in respiratory complex assembly. Scatter plots and correlation coefficients are in Figure S3.

The second group consists of 11 genes known to be associated with the actin cytoskeleton, including *AIM21* as described in Results. The biochemical functions of the other 10 proteins with respect to actin have been previously described [68]–[71], but they had no previously known mitochondrial roles. For example, Cap2p has been characterized *in vitro* to bind the barbed ends of actin filaments and prevent further polymerization [72], but it has not been previously implicated in mitochondrial transmission. Interestingly, many of this specific subgroup of actin-associated proteins have also been implicated in actin/membrane interactions for endocytic trafficking [73],[74]. This raises the intriguing possibility that these proteins have specialized in interactions between actin and intracellular membranes.

### Extensions to Other Biological Systems

Our general approach can be successfully extended to other processes beyond mitochondrial biogenesis in yeast and to other organisms. We have applied our computational ensemble [26],[28],[29] to 388 other processes in *Saccharomyces* with promising results (Figure S2), and we report functional predictions for these processes (Dataset S2). Computational methods have also been successfully applied in other organisms with readily available genomic data collections [1],[2],[75], and the iterative nature of our approach may be particularly useful in higher eukaryotes where current functional knowledge is relatively sparse. Directing assays with computational predictions is especially attractive in higher organisms where time and resource commitments are prohibitive.

These results demonstrate the utility of employing computation to direct quantitative, functionally definitive assays. Here, we have used this technique to newly confirm the involvement of 109 proteins in the process of mitochondrial biogenesis in *S. cerevisiae* by assaying the frequency of petite colony formation. A subset of these proteins was also characterized using growth profiling and immunofluorescence microscopy, revealing participation in specific sub-processes of mitochondrial biogenesis. In particular, *AIM21* was shown to be required for proper mitochondrial motility, a discovery which would have been difficult to make without specifically targeted computational predictions. As these techniques can be naturally extended to additional organisms and processes, close integration of computational function prediction with experimental work in other biological systems promises to quickly direct experimenters to novel facets of their areas of interest.

## Materials and Methods

### Petite Frequency Assay

This protocol is adapted from the original petite frequency [31] and tetrazolium overlay [76] assays. For each mutant strain tested, we grew several replicates of the strain for 48 hours in liquid YP Gycerol at 30°C [77]. Strains able to grow on glycerol were diluted and plated for single colonies on YPD plates, which releases the requirement for functional mitochondrial DNA. Thus, as colonies formed, cells without functional mitochondrial DNA were generated. When the colony is fully formed it is a mixture cells with functional mitochondrial DNA and cells without functional mitochondrial DNA. We measured this ratio by re-suspending a colony and plating a dilution of this re-suspension such that 100–300 colonies are formed on a YPD plate. By overlaying with soft agar containing tetrazolium, colonies with functional mitochondria were stained red, while colonies without functional mitochondria remained white. The final mixture for agar overlay contains: 0.2% 2,3,5 -triphenyltetrazolium chloride (Sigma T8877), 0.067 M sodium phosphate buffer pH 7.0 and 1.5% bacto agar. The ratio of white colonies to total colonies gives the petite frequency. Eight independent petite frequencies (biological replicates) were measured for each strain tested. The distribution of these frequencies was compared to the frequency of petite generation in wild-type yeast. Strains identified as having the altered mitochondrial transmission phenotype in this assay exhibit at least a 20% change in petite frequency from wild type, and have a p-value of less than 0.05 when comparing the petite frequency distributions of that strain to the wild-type petite frequency distribution, using a Mann-Whitney U test.

### Computational Prediction Ensemble Methodology

The three computational systems employed in our study were bioPIXIE [26],[27], MEFIT [28], and SPELL [29]. Each was used to analyze genes involved in the GO biological process ‘mitochondrion organization and biogenesis’ (GO:0007005). All methods were initially trained and/or evaluated using the 106 annotations to this process as of April 15^th^, 2007. Detailed descriptions of these methods can be found in their respective publications.

### Identification of Additional Control Genes with Literature Evidence

42 of our initial computational predictions had strong literature evidence for involvement in mitochondrial biogenesis and inheritance and were determined to be “under-annotated” – meaning that they already had strong literature evidence for their involvement in mitochondrial organization and biogenesis, but were not yet annotated to the corresponding GO term. These 42 genes, along with 6 genes already annotated, were included as our positive control set of 48 genes. In most of these 42 cases the information was already curated by SGD in the form of annotations to other GO terms, such as ‘integral to the mitochondrial membrane’ or ‘mitochondrial protein import.’ In addition to these 42 genes, we identified an additional 95 genes that we believe have enough literature evidence to warrant their inclusion in this process without further laboratory testing, for a total of 137 “under-annotated” genes. All 137 of these genes were included in the training set for our second iteration of computational predictions.

### Selection of Prediction Candidates for Experimental Testing

Novel candidates for laboratory evaluation were chosen on the basis of both the three individual computational approaches as well as the ensemble of their predictions. We limited ourselves to consider only those genes with viable knockouts available in the heterozygous deletion collection [55]. Furthermore, we chose to evaluate predictions to both genes with no previously known function as well as genes known to be involved in a biological process other than mitochondrial inheritance and biogenesis. We chose the 20 most confident genes of unknown function and the 20 most confident genes with existing annotations to other biological processes from each of the three individual methods for validation. Due to overlaps between the predictions of each method, there were 87 genes in this group, however, 20 of these genes we determined to be “under-annotated” and were tested as positive controls, leaving 67 genes used as novel candidates without any prior literature evidence. We then chose an additional 74 genes from the ensemble list of predictions with no previous literature evidence to arrive at our total of 141 test candidates in our first round of laboratory evaluation.

### Iterative Re-Training, Prediction, and Verification

After our first round of testing, 82 of the 141 novel predictions were discovered to have involvement in mitochondrial inheritance and biogenesis. Combined with the original 106 annotated genes and the 137 genes identified as “under-annotated,” this results in a total of 325 genes. Each of the three computational methods was re-applied using this updated training set of 325 genes and the same procedure was used to form an updated ensemble list of predictions. We selected for laboratory investigation the 52 genes with the highest confidence from the updated results that were not previously tested. The petite frequency assay was used, and an additional 17 genes demonstrated a significant phenotype.

### Double Mutant Construction and Testing

Deletion alleles marked with the ClonNAT resistance gene (rather than the G418 KanMX resistant marker) were prepared for the four tested strains (*aim17*Δ, *rvs167*Δ, *tom6*Δ, and *ehd3*Δ). A ClonNAT marked *ura3*Δ allele was prepared as a control (all other strains contained a *ura3*Δ allele. These ClonNAT resistant strains contained the Magic Marker reporter [34] as well as *can1*Δ and *lyp1*Δ mutations to reinforce haploid selection. These five strains were crossed to a set of deletion strains marked with the G418 resistant KanMX marker, and diploids were selected on YPD-G418-ClonNAT. The diploids were then sporulated as described for our single mutant assays, except that double mutants were selected on media containing G418 and ClonNAT, and three controls were isolated for each sporulation: G418 resistant mutant, ClonNAT resistant mutants, and wild-type strains. The petite frequency assay was applied to these double mutant strains as described above. Phenotypic calls were determined for the double mutants based on the significance of the difference between the distributions of petite frequencies of the double strain versus both of the corresponding single strains. If the FDR corrected joint Wilcoxon rank sum p-value of both of these comparisons was <0.05, and the distribution of the double mutant strain was significantly different from wild type, then we scored the double mutant as significantly altered.

### Yeast Strains and Media

All *S. cerevisiae* strains used in this study are descended from the S288C derivative used for the deletion consortium project [55]. Methods for individual mutant manipulation are described below. Standard methods for media preparation were used as previously described [77].

### Deletion Set Manipulation

The Magic Marker heterozygous yeast deletion set [34] was pinned from glycerol stocks onto enriched sporulation agar as described [78]. Single colonies developing on these random spore plates were re-struck for single colonies on the same medium and tested for presence of the G418 resistant KanMX marker [34] to identify the spore as wild-type or a deletion mutant. Single colonies that grew from this re-streaking process were picked and arranged in 96 well plates containing YPD. Each set of strains for a given candidate gene of interest were placed in a single column (1–12 of a 96 well plate); mutant isolates were placed in the first six wells (A–F) and sister wild type isolates were placed in wells G and H. These 96 well plates were glycerol stocked.

### Growth Rate Assay

Strains were measured for their ability to grow in both respiratory (2% glycerol as carbon source) and fermentative (2% glucose as carbon source) conditions in minimal media supplemented for auxotrophies. Cultures were grown at 30°C. Growth curves were generated in a 96-well plate format (described above in “Deletion Set Manipulation”) that tests 12 mutants per run. For each mutation tested, 6 independent deletion mutants of that gene were grown in separate wells. Twenty-four replicate wild-type strains were also present in each 96-well plate format. Plates were grown and measured using a Tecan GENios plate incubator and reader, which recorded densities every 15 minutes for 24 hours for glucose cultures and 48 hours glycerol cultures. The raw growth data are available in Dataset S1.

### Growth Rate Data Processing

Growth rates were derived from these curves by using Matlab to fit an exponential model:
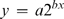
For each well, this model was fit over the entire curve, the first 2/3, and the first half; whichever yielded the best fit was used in downstream analysis (to avoid plateau effects and to model only exponential growth). Wells with an adjusted R^2^<0.9 were marked as non-growing, and growth rates for the remaining wells were determined by subtracting the row, column, and plate means for each well from the exponential parameter *b*, yielding a rate *b*' for each well. These *b*' parameters for each mutant strain were tested for significance against the total wild type population (excluding non-growing wells) using a Mann-Whitney U test. Significance was only considered for *b*' parameters indicating a slower growth rate than wild-type.

To detect colonies growing exponentially but with significant differences in fitness, smoothed maximum densities *d* were also calculated for all wells, consisting of the average of the optical density readings for the last five time points in each growth curve. From these, plate, row, and column averages were subtracted from each well, generating adjusted maxima *d*'. Mutants which did not double in optical density at least once (i.e. where *d*' was less than twice the baseline optical density) were considered to be non-growing. The remaining *d*' values for each mutant were again compared with the wild type values (excluding non-growing wells) using a Mann-Whitney U test. Significance was only considered for *d*' parameters indicating a lower saturation density than wild-type. Combined with the exponential rate tests, this assigned each mutant phenotypes in rich media and in glycerol of no effect, no growth, or significant sickness.

In either assay, mutants with inconsistent results (disagreement among more than one of the six replicates) were deemed inconclusive and marked as “mixed”. Phenotypes were never assigned based on such mixed phenotypes. For a mutant to be classified as having a respiratory growth defect, that defect was required to be specific to the glycerol media (i.e. no phenotype in glucose). If the mutant grew slowly in both glycerol and rich media, then it was not considered to have a defect in respiration.

### Immunofluorescence

Yeast immunofluorescence was carried out using standard methods [77]. Briefly, strains were grown to exponential phase in synthetic complete medium, and fixed in freshly prepared formaldehyde for 1 hour at 30 degrees. (Mutant strains were isolated from the Magic Marker deletion set as described above; FY4 was used as a wild type strain for comparison.) Cells were washed, digested with Zymolyase and attached to polyethyleneimine-coated coverslips. Cells were blocked with BSA, and exposed to an anti-porin antibody (Invitrogen, A-6449) and a guinea pig anti-yeast actin antibody [79]. Secondary antibodies were Alexa 488-conjugated goat anti-guinea pig (Invitrogen, A-11073) and Alexa 555-conjugated goat anti-mouse (Invitrogen A-31621). Coverslips were mounted in PBS/glycerol/phenlyenediamene. Microscopy was performed on a Perkin Elmer RS3 spinning disk confocal microscope with a 100× objective. Exposures were 1 ms per slice, and Z-stacks were taken with a 0.15 um spacing, and images were deconvoluted and assembled into 3D volumes using Volocity (Improvision).

### F-Actin Staining

Phalloidin staining was performed according to Methods in Yeast Genetics [77]. Briefly, strains were growth to exponential phase in synthetic complete medium, and fixed in formaldehyde (Electron Microscopy Sciences 15712-5) for one hour. F-actin was stained using Alexa 488 conjugated phalloidin (Invitrogen, A12379). Cells were deposited on polyethyleneimine-coated coverslips and mounted in PBS/glycerol/phenlyenediamene. Slides were imaged and processed as for immunofluorescence.

### Integration of Mitochondrial GFP into Deletion Strains

The NatMX cassette was cut from pAG25 [80] using NotI and ligated into the EagI site of pYX122-mtGFP, which expresses a mitochondrially targeted GFP (directed by the Su9 peptide) under the control of the triose phosphate isomerase promoter [81]. This construct was used as a template to PCR amplify the NatMX-mtGFP cassette using primers with 40 bp homology to target the cassette for integration at the dubious ORF, YDL242W. This integration was performed in the strain Y5563 to create ACY50 (strain list, Table S1). ACY50 was then mated to the Magic Marker yeast deletion set [34] and selected for haploid deletion mutants carrying the cassette as described [78].

### Mitochondrial Tracking Microscopy

Exponential phase cultures of *S. cerevisiae* in Yeast synthetic complete media (YSC) were plated onto glass slides with an agarose bed growth chamber made of low melt agarose and YSC media. The slides were covered with a cover slip and sealed using VALAP [82]. Cells were then imaged using a Perkin Elmer RS3 spinning disk confocal microscope with a 100× objective. Images of mitochondrial GFP fusions were taken using a laser emitting at 488 nm at 100% power with an exposure of 1 sec. Phase contrast images were taken using an exposure of 3 ms. For all images, 2×2 binning was used and gain was set to 255. For each field of view, both an initial z-stack of images and a time course were taken. Each z-stack was taken at intervals of 0.2 µm through the entire depth of the cells. The time course was taken in a single focal plane for two minutes at 1 frame per second. (Raw imaging files are available upon request.)

To determine the frequency of sustained mitochondrial movement resulting from Brownian motion or other passive processes [83], sustained mitochondrial movement was also measured in the presence of the metabolic inhibitors sodium azide and sodium fluoride. These inhibitors were added to the YSC agarose used for imaging; 10 mM concentrations of these inhibitors were compared to a control of 10 mM NaCl.

### Image Processing: Frequency of Sustained Mitochondrial Movement

To measure mitochondrial motility *in vivo*, individual mitochondrial tips were tracked through each frame of a 2 minute time course using the Manual_Tracking plugin for ImageJ. Image files were randomly coded with numbers so that the identity of each imaged strain was not known to the investigator performing image tracking. Mitochondria tips for tracking were identified using the Z-stacks (which avoids selection of tubules that appear to be tips because other sections are out of plane). These Z-stacks were assembled into a Z-projection and merged with the phase contrast image using ImageJ to permit identification of budded cells. The selection criteria for mitochondrial tips were that the tip is initially present in the mother cell of a budded cell. In cases where both termini of a mitochondrion were available for tracking, the tip closer to the daughter cell was selected. The position of the bud neck was set as a reference and the position of a mitochondrial tip in the mother cell was plotted for each of 120 frames. The distance from the mitochondrial tip to the bud neck was calculated at each frame (in our imaging hardware, each pixel corresponded to 0.15 µm). Mitochondrial movement in each frame was then calculated by subtracting the distances to the bud neck in two consecutive frames and dividing by the time interval of 1 second. The tracking data are available in Table S8. Sustained mitochondrial movement events consisting of 3 consecutive frames of motion towards (anterograde) or 3 consecutive frames of motion away (retrograde) from the bud were identified using a custom Perl script [54]. The number of these sustained movement events per minute was calculated.

### Ensemble Predictions for Additional Biological Processes

In addition to the computational predictions used to study mitochondrial biogenesis and inheritance in this study, we have applied the same prediction techniques to many additional biological processes in *S. cerevisiae*. We used each of our three computational methods to predict gene functions for 387 additional biological processes in the same manner as described above. The full prediction lists for all of these processes are available in Dataset S2. In order to demonstrate that these methods are able to capture information about these processes, we have also calculated the average precision (*AP*) of the cross-validated results as:
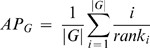
where *G* is a group of genes known to be involved in a process, and *rank_i_* is the rank order of gene *i* in the prediction results. A graph of the average precisions for all 388 processes (including mitochondrial organization and biogenesis, highlighted in red) is shown in Figure S2.

### Human Ortholog Identification

Orthology between yeast and human genes was based on orthologous clusters in the Homologene [84], Inparanoid [85], and OrthoMCL [86] databases as of June 2007. Each of these uses a published algorithm for determining clusters of orthologous genes, i.e. groups of genes thought to be conserved and perform near-identical functions in different organisms. We first took the union of these databases as applied to a core set of diverse organisms (yeast, human, mouse, fly, and worm), considering a gene pair to be orthologous if declared so by any of the three databases. This resulted in a set of unified orthologous clusters, from which we eliminated any cluster containing more than 50 genes. This resulted in 14,528 clusters spanning 61,702 genes in the five organisms, and from this set we report here the human orthologs of *S. cerevisiae* genes in our study.

### Human Ortholog Disease-Related Gene Identification

Disease related human orthologs were determined based on the manual curation of the Online Mendelian Inheritance in Man (OMIM) resource [87], and the automated text mining available through GeneCards [88]. We considered all of the OMIM curations valid, while we required at least 2 independent publication citations in GeneCards for a disease relation to be valid.

### Localization Determination

Both mitochondrial and actin localization was based on the Gene Ontology cellular component curation. For mitochondrial localization curation to the term GO:0005739: mitochondrion was used. In addition six genes were marked as computationally predicted to the mitochondrion based on the study by Prokisch et al. [50]. For actin localization curation to the term GO:0015629: actin cytoskeleton was used.

## Supporting Information

Figure S1 Phalloidin staining of yir003wD_ puf3D_ and wild-type strains. F-actin was stained using Alexa-488 labeled phalloidin. Actin polarization appears normal in the mutant strains, because actin patches are polarized to daughter cells, and unbudded cells have few actin patches. Also, actin cables appear normal in the mutant strains.
(1.96 MB PDF)

Figure S2 Average precision of our ensemble applied to 388 biological processes. We applied the same ensemble of computational methods to 387 additional biological processes in the same manner that we generated candidate predictions for mitochondrial biogenesis. This figure shows the cross-validated average precision of this ensemble for all of these processes. The red arrow indicates the mitochondrial biogenesis process. The full prediction lists for these processes is available in Dataset S2.
(0.09 MB PDF)

Figure S3 Scatter plots and correlations between petite frequency data and glycerol growth phenotypes. Strong correlation is not observed between the petite frequency phenotypes and the glycerol growth phenotypes. We compared the petite frequency phenotypes to both the glycerol doubling time and the saturation density (plots A and B). We also compared subsets of the petite frequency data to the glycerol growth phenotypes. In plots C and D, mutants with a decreased petite frequency phenotype were compared to the glycerol growth phenotypes. In plots E and F, mutants with an increased petite frequency phenotype were compared to the glycerol growth phenotypes. As we note in the discussion section of the main paper, the processes of respiration and of mitochondrial transmission are overlapping but distinct biological functions. While they are clearly related in that both require operational mitochondria, the ability of the cell to cope with a partial defect in transmission often leaves that cell with enough functional mitochondria to perform respiration. Thus, particularly in a quantitative setting, these two phenotypes are not necessarily correlated.
(0.23 MB PDF)

Table S1 Strains used in this study.
(0.04 MB XLS)

Table S2 Summary list of genes tested in this study.
(0.14 MB XLS)

Table S3 Gold Standards used for 1st and 2nd iteration computational predictions.
(0.04 MB XLS)

Table S4 Computational prediction lists from the 1st iteration for genes with mitochondrial function.
(4.27 MB XLS)

Table S5 Computational prediction lists from the 2nd iteration for genes with mitochondrial function.
(4.35 MB XLS)

Table S6 Raw petite frequency single mutant data.
(0.15 MB XLS)

Table S7 Raw petite frequency double mutant data.
(0.06 MB XLS)

Table S8 Raw data for mitochondrial motility assays.
(0.04 MB XLS)

Dataset S1 Raw respiratory growth data. A zip file containing 48 tab-delimited text files containing the raw data from the Tecan GENios reader. Additionally, a readme text file and plate key is contained in the zip file. The plate key uses our internal reference id for each mutant (internal reference id is matched with common and systematic gene names in Table S2). Raw optical density measurements from Tecan GENios plate incubator and reader. Wells are numbered in increasing order from upper left to lower right and can be converted to our internal identifiers using the included key (complete plate key). Not all original plates were assayed for growth curves, and there are two replicates of plate 20.
(1.82 MB ZIP)

Dataset S2 Top 100 novel ensemble predictions for additional yeast biological processes. A zip file containing 387 tab-delimited text files of the ensemble predictions for the indicated Gene Ontology Biological Process Term.
(21.70 MB GZ)
