# Supplementary material for: Computationally Driven, Quantitative Experiments Discover Genes Required for Mitochondrial Biogenesis
Source: PLoS Genet. 2009 Mar 20;5(3):e1000407. doi: 10.1371/journal.pgen.1000407 (PMC2648979; doi:10.1371/journal.pgen.1000407)

Figure S1. Phalloidin staining of yir003wD, puf3D, and wild-type strains.

F-actin was stained using Alexa-488 labeled phalloidin. Actin polarization appears normal in the mutant strains, because actin patches are polarized to daughter cells, and unbudded cells have few actin patches. Also, actin cables appear normal in the mutant strains.

A) wild-type (FY4)

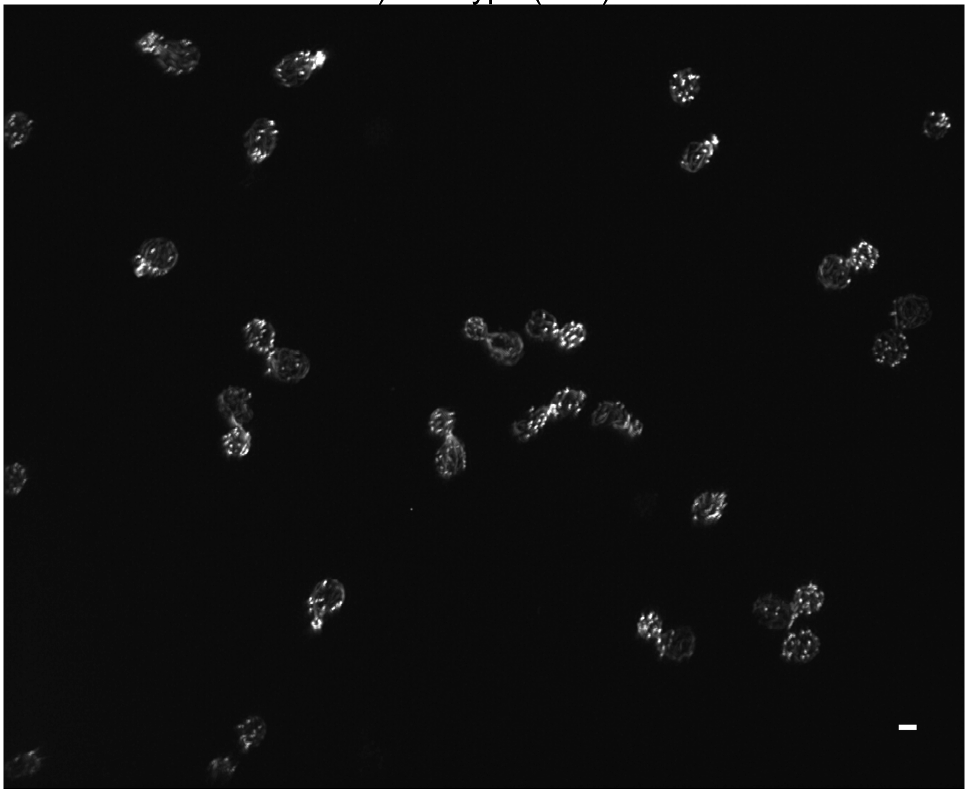

B) *puf3Δ*

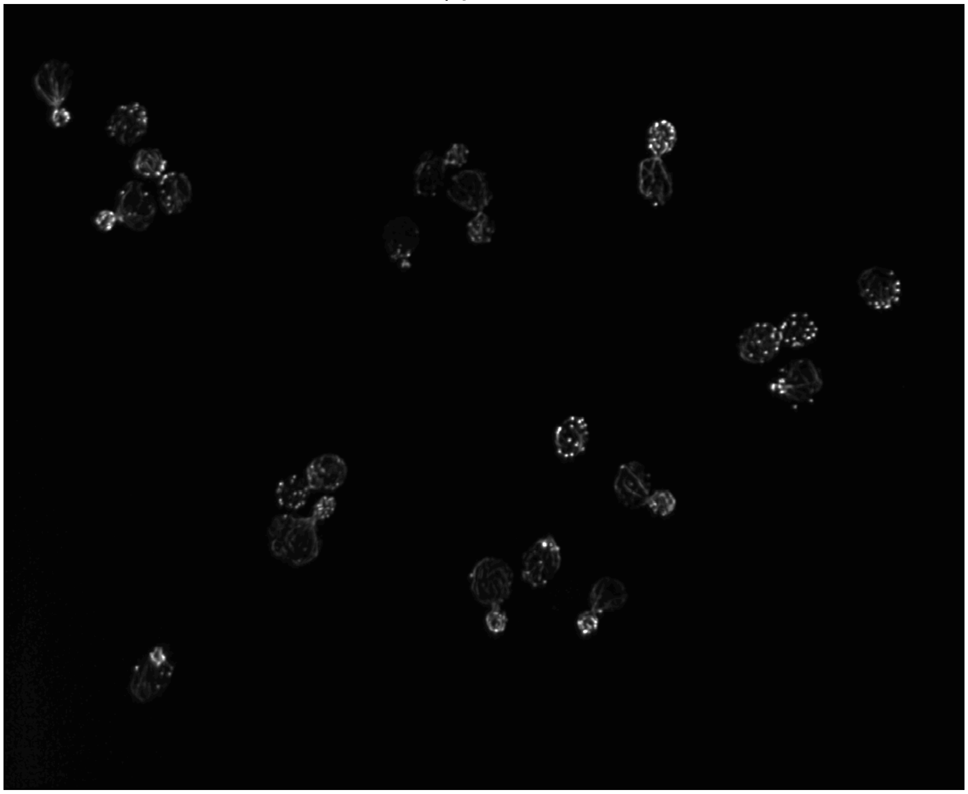

C) *aim21Δ*

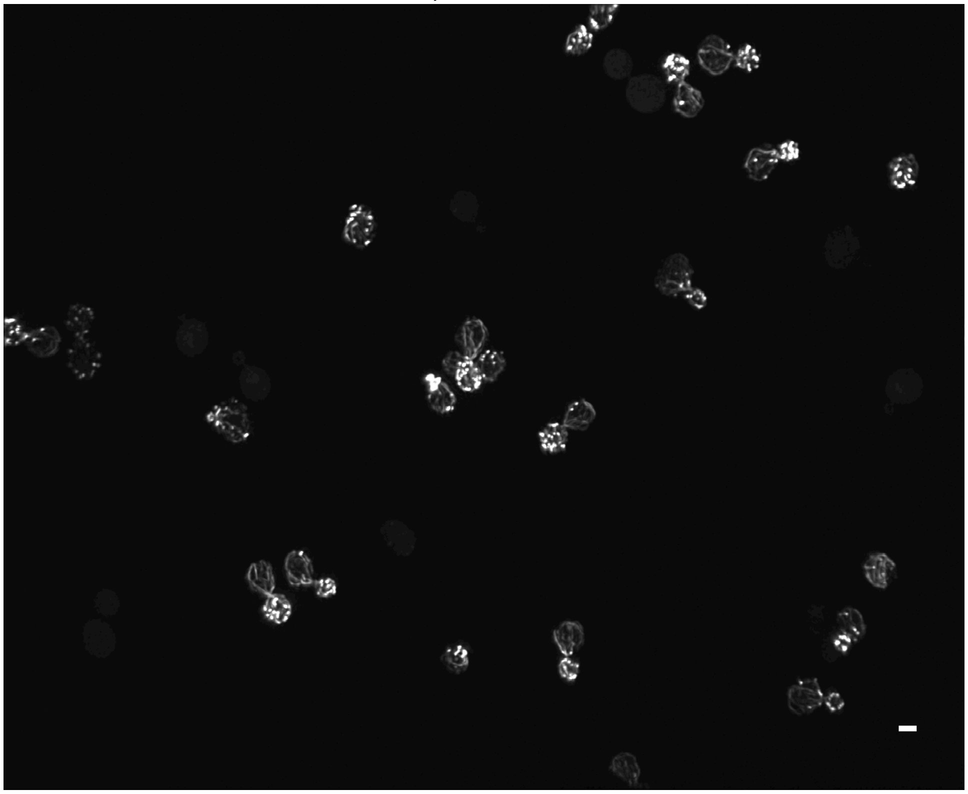

Supplement: Figure S1 — Phalloidin staining of yir003wD_ puf3D_ and wild-type strains. F-actin was stained using Alexa-488 labeled phalloidin. Actin polarization appears normal in the mutant strains, because actin patches are polarized to daughter cells, and unbudded cells have few actin patches. Also, actin cables appear normal in the mutant strains. (1.96 MB PDF) [file pgen.1000407.s001.pdf]
