# Supplementary material for: Computationally Driven, Quantitative Experiments Discover Genes Required for Mitochondrial Biogenesis
Source: PLoS Genet. 2009 Mar 20;5(3):e1000407. doi: 10.1371/journal.pgen.1000407 (PMC2648979; doi:10.1371/journal.pgen.1000407)

Figure S2. Average precision of our ensemble applied to 388 biological processes.

We applied the same ensemble of computational methods to 387 additional biological processes in the same manner that we generated candidate predictions for mitochondrial biogenesis. This figure shows the cross-validated average precision of this ensemble for all of these processes. The red arrow indicates the mitochondrial biogenesis process. The full prediction lists for these processes is available in Table S9.

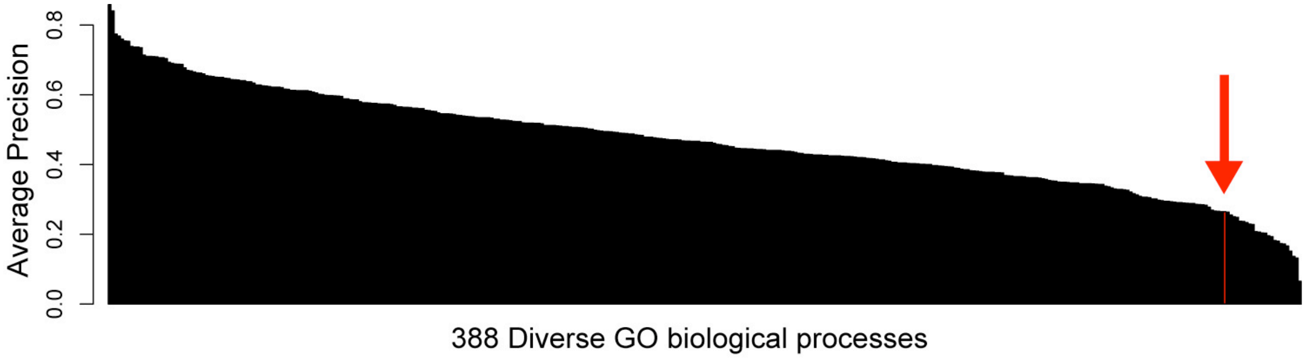

Supplement: Figure S2 — Average precision of our ensemble applied to 388 biological processes. We applied the same ensemble of computational methods to 387 additional biological processes in the same manner that we generated candidate predictions for mitochondrial biogenesis. This figure shows the cross-validated average precision of this ensemble for all of these processes. The red arrow indicates the mitochondrial biogenesis process. The full prediction lists for these processes is available in Dataset S2. (0.09 MB PDF) [file pgen.1000407.s002.pdf]
