# Supplementary material for: Computationally Driven, Quantitative Experiments Discover Genes Required for Mitochondrial Biogenesis
Source: PLoS Genet. 2009 Mar 20;5(3):e1000407. doi: 10.1371/journal.pgen.1000407 (PMC2648979; doi:10.1371/journal.pgen.1000407)

Figure S3. Scatter plots and correlations between petite frequency data and glycerol growth phenotypes.

Strong correlation is not observed between the petite frequency phenotypes and the glycerol growth phenotypes. We compared the petite frequency phenotypes to both the glycerol double-time and the saturation density (plots A and B). We also compared subsets of the petite frequency data to the glycerol growth phenotypes. In plots C and D, mutants with a decreased petite frequency phenotype were compared to the glycerol growth phenotypes. In plots E and F, mutants with an increased petite frequency phenotype were compared to the glycerol growth phenotypes. As we note in the discussion section of the main paper, the processes of respiration and of mitochondrial transmission are overlapping but distinct biological functions. While they are clearly related in that both require operational mitochondria, the ability of the cell to cope with a partial defect in transmission often leaves that cell with enough functional mitochondria to perform respiration. Thus, particularly in a quantitative setting, these two phenotypes are not necessarily correlated.

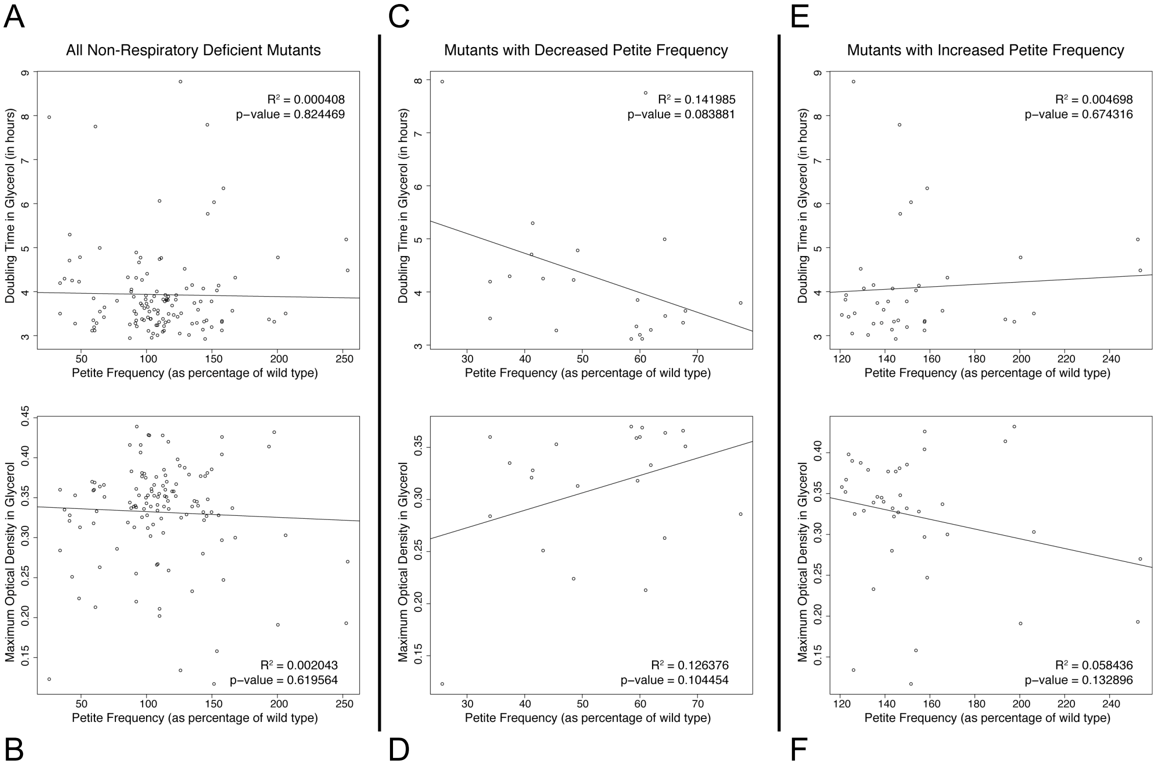

Supplement: Figure S3 — Scatter plots and correlations between petite frequency data and glycerol growth phenotypes. Strong correlation is not observed between the petite frequency phenotypes and the glycerol growth phenotypes. We compared the petite frequency phenotypes to both the glycerol doubling time and the saturation density (plots A and B). We also compared subsets of the petite frequency data to the glycerol growth phenotypes. In plots C and D, mutants with a decreased petite frequency phenotype were compared to the glycerol growth phenotypes. In plots E and F, mutants with an increased petite frequency phenotype were compared to the glycerol growth phenotypes. As we note in the discussion section of the main paper, the processes of respiration and of mitochondrial transmission are overlapping but distinct biological functions. While they are clearly related in that both require operational mitochondria, the ability of the cell to cope with a partial defect in transmission often leaves that cell with enough functional mitochondria to perform respiration. Thus, particularly in a quantitative setting, these two phenotypes are not necessarily correlated. (0.23 MB PDF) [file pgen.1000407.s003.pdf]
